# Intraepidermal Nerve Fiber Density as an Indicator of Neuropathy Predisposition: A Systematic Review with Meta-Analysis

**DOI:** 10.3390/diagnostics15111311

**Published:** 2025-05-23

**Authors:** Alexandros Samolis, Theodore Troupis, Constantinus Politis, Nikos Pantazis, George Triantafyllou, George Tsakotos, Thomas Tegos, Nikolaos Lazaridis, Konstantinos Natsis, Maria Piagkou

**Affiliations:** 1 Department of Anatomy, School of Medicine, Faculty of Health Sciences, National and Kapodistrian University of Athens, 11527 Athens, Greece; alexsamolis@me.com (A.S.); ttroupis@gmail.com (T.T.); georgerose406@gmail.com (G.T.); gtsakotos@gmail.com (G.T.); 2 OMFS-IMPATH Research Group, Department of Imaging and Pathology, Faculty of Medicine, Katholieke Universiteit Leuven, 3000 Leuven, Belgium; stan@politis.be; 3 Department of Hygiene, Epidemiology and Medical Statistics, Medical School, National and Kapodistrian University of Athens, 11527 Athens, Greece; npantaz@med.uoa.gr; 4 Neurological Department, Aristotle University of Thessaloniki, AHEPA General Hospital, 54634 Thessaloniki, Greece; ttegos@auth.gr; 5 Department of Anatomy and Surgical Anatomy, Faculty of Health Sciences, Aristotle University of Thessaloniki, 54124 Thessaloniki, Greece; nikolaosmd@hotmail.com (N.L.); natsis@auth.gr (K.N.)

**Keywords:** intraepidermal nerve fiber density, neuropathy, skin biopsy, small fiber neuropathy, healthy, meta-analysis

## Abstract

**Background/Objectives**: Skin spot biopsy is the gold standard for diagnosing small fiber neuropathy. A systematic approach to intraepidermal nerve fiber density (IENFD) was conducted to estimate its value precisely in healthy and neuropathic subjects, independent of the neuropathy disease. The findings will serve as a guidance model for IENFD as an indicator of neuropathy predisposition. It was also investigated how IENFD was influenced by age, gender, and neuropathy. **Methods**: A systematic search of PubMed, Web of Science, and ScienceDirect was conducted to identify clinical studies from 1997 to 2022 concerning IENFD in healthy and neuropathic adult populations. Data were retrieved from longitudinal cohort studies, including 5–188 healthy and 6–40 neuropathic patients. Multilevel meta-regression was employed to assess associations between the anatomical region, mean patient age, and male/female ratio with IENFD. This method accounted for correlations between multiple outcomes from the same survey, offering a more nuanced analysis than standard meta-regression. **Results**: In the healthy population, the estimated (95% CI) IENFD values (fibers/mm) were 21.4 (19.9, 22.9) in the thigh, 17.7 (15.3, 20.1) in the forearm, 12.9 (11.8, 14.0) in the distal leg, 11.3 (6.1, 16.5) in the fingers, and 6.5 (4.4, 8.6) in the toes. The corresponding estimates in the neuropathic population were 17.2 (15.2, 19.2) in the thigh, 6.3 (2.3, 10.2) in the forearm, 5.1 (3.8, 6.4) in the distal leg, and 2.0 (0.0, 5.7) in the toes. In a healthy population, IENFD decreased with aging by 1.35 fibers/mm every 5 years (*p* < 0.001). Gender dimorphism in IENFD existed, with females showing higher values in the distal leg (13.6–10.5) compared to males (9.3–7.2). **Conclusions**: The systematic study and meta-analysis integrate evidence of IENFD in skin biopsies. This analysis reconciles findings from various methodologies and populations over two decades. Meta-regression techniques address variability due to biopsy site, fixation protocols, immunohistochemical markers, and demographics. To reduce future study heterogeneity, using the thigh is advisable as it shows the least variability. Additionally, standardizing the biopsy site internationally will ensure comparability. These findings urge further investigation into IENFD changes in neurodegenerative diseases and whether IENFD can be a reliable prognostic marker for neuropathy diagnosis.

## 1. Introduction

Skin spot biopsy is the gold standard for diagnosing small fiber neuropathy (SFN) [1]. Over the last twenty years, efforts have been made to measure intraepidermal nerve fiber density (IENFD) through quantitative analysis in various anatomical regions, including the forearm, thigh, and distal leg. Skin biopsy is a minimally invasive technique that allows for the identification of nerve fiber populations based on their targets. In patients with SFN symptoms, assessing IENFD is the gold standard for diagnosis, while quantifying sudomotor, pilomotor, and vasomotor nerve fibers evaluates autonomic involvement. These parameters can be re-evaluated over time to monitor disease progression and treatment effectiveness. Myelinated fibers and their receptors can also detect early signs of “dying back” neuropathy when nerve conduction studies are normal. Additionally, dermal myelinated fiber morphometry offers insights into the mechanisms of inherited and acquired large fiber neuropathies (LFNs) [2]. The loss of IENFs serves as the primary morphological indicator and the sole objective assessment that confirms a suspicion of SFN. Alongside IENF loss, specific morphological changes, including varicosity [3] and irregular nerve distributions in the epidermis [4], can indicate potential fiber loss. Furthermore, a skin biopsy can distinguish between length-dependent neuropathy and non-length-dependent ganglionopathy by evaluating the IENFD ratio from the distal and proximal lower limb sites [5]. This characteristic can assist clinicians in the screening process for SFN.

Immunohistochemistry (IHC) with rabbit antiserum against human protein gene product (PGP) 9.5 has opened new avenues for detecting free sympathetic nerve fibers (SNFs) [6,7]. Kim et al. [8] highlighted the high accuracy of three-dimensional microscopy compared to conventional bright-field microscopy for quantifying free SNFs. The assessment of IENFD is a cornerstone diagnostic tool in SFN and autonomic disorders. Two primary staining methods are used: IHC and immunofluorescence (IF). Research shows that IF generally provides better sensitivity, clarity, and reproducibility, especially for subtle fiber loss. Each technique has specific strengths and limitations for different situations. The gold standard for evaluating IENFD is IF due to its high-resolution detection of unmyelinated SNFs, which is crucial for diagnosing SFN and peripheral nerve research. IF allows for better visualization, co-labeling, and is more suitable for digital image analysis than traditional IHC. Therefore, IF is preferred in research and diagnostics in neuropathology [9].

We extensively searched electronic databases and identified clinical studies that describe significant heterogeneity due to interpopulation and intrapopulation differences. Heterogeneity is reported among different human body locations (upper and lower limbs) and in healthy subjects versus those with neuropathic conditions. A decreased IENFD is associated with pathological entities, including the following neuropathies: small fiber neuropathy, diabetic neuropathy, HIV neuropathy (symptomatic and asymptomatic), Friedreich’s ataxia, vasculitis, idiopathic neuropathy, sensory ganglionopathy, symmetrical sensory polyneuropathy, sensory neuronopathy in systemic lupus erythematosus, neuropathy from chronic inflammatory demyelination, neuropathy in sarcoidosis and axonal swelling, and Charcot-Marie-Tooth disease type 1A [3,6,10,11,12,13,14,15,16,17,18,19,20,21,22,23,24,25,26,27,28,29,30,31]. Although IENFD decrease correlates with neuropathy, the lack of estimated IENFD values in healthy populations impedes direct comparisons with several types of neuropathies.

In the current meta-analysis, we systematically approached the IENFD to extract an estimated reference value in a population. Our findings could serve as a guidance model for further use of IENFD as an indicator for assessing neuropathy predisposition. Moreover, we investigated how the estimated IENFD value was related to age, gender, and neuropathy.

## 2. Materials and Methods

### 2.1. Search Strategy

We systematically searched three online databases (PubMed, Web of Science, and ScienceDirect) to identify clinical studies investigating IENFD in adult (healthy and neuropathic) populations. An extensive literature search was conducted by three independent investigators, using the keywords: “epidermal nerve fiber density” or “epidermal nerve fiber density” and “distal leg” or “epidermal nerve fiber density” and “thigh” or “epidermal nerve fiber density” and “fingers” or “epidermal nerve fiber density” and “forearm” or “epidermal nerve fiber density” and “immunofluorescence” or “epidermal nerve fiber density” and “neuropathy” or “epidermal nerve fiber density” and “pathology” or “skin biopsy” and “small fiber” or “sympathetic nerve fiber” and “PGP 9.5” or “autonomic nervous system” and “PGP 9.5” or “sensory small fiber” and “PGP 9.5”. The keywords were identified in the paper’s title, abstract, or full text. Following the acquisition of the full texts, we performed a further search to identify other potentially eligible articles that may not have been identified in our search of the online databases. The protocol was not registered in any database.

### 2.2. Inclusion Criteria

We reviewed clinical studies that involved healthy adult (male and female) subjects, as well as patients with neuropathy resulting from various diseases affecting the upper and lower limbs. All included studies calculated the IENFD after a skin spot biopsy [32] from various anatomical regions, such as the forearm, finger, thigh, distal leg, and toe. The skin spot biopsies were taken from the anterior or the posterior surface, or the proximal or distal examined areas. They were grouped by anatomical region: forearm, fingers, thigh, distal leg, and toes. All the included studies used a contiguous IF technique. IF is the gold standard for evaluating IENFD due to its superior ability to detect small, unmyelinated fibers with high resolution and contrast. It allows for accurate and reproducible quantification, which is critical in diagnosing SFN. IF provides superior visualization, enables co-labeling, and is more suitable for digital image analysis than traditional IHC for evaluating IENFD. This makes IF the preferred method, particularly in research and diagnostic neuropathology [9].

### 2.3. Statistical Analysis

Data concerning the IENFD measurements were recorded as means ± standard deviations (SD). In the study by Bakkers et al. [33], overall mean values and SDs were calculated based on the relative values per age group, assuming normal distributions and using Monte Carlo techniques. In the study by Hermann et al. [27], means ± SD from symptomatic and asymptomatic neuropathic patients were combined using standard formulas. In the Hoitsma et al. [25] study, means ± SD were estimated based on minimum and maximum values employed in the formulas outlined in Hozo et al. [34]. Data from all studies were summarized through medians and interquartile ranges (IQRs) per anatomical location (site) of the spot biopsies to determine the dispersion of the measured parameters. Considering that the IENFD values are differentiated per spot biopsy, we also calculated the coefficient of variation (CV), which represents the SD as a percentage of the mean. Lower CV values indicate higher repeatability of measurements for specific anatomical regions, assuming that study populations are similar and do not systematically differ in dispersion. Initial exploratory analyses were based on standard meta-analysis techniques: pooled estimates and the corresponding 95% confidence intervals (CIs) per anatomical region of measurement, and overall, they were obtained for neuropathic and healthy individuals. The estimates were derived using random-effects meta-analysis (Der Simonian-Laird method). The corresponding forest plots are presented. We used a random-effects approach due to the high heterogeneity among studies. To identify sources of variability, we employed meta-regression techniques. More specifically, formal inference was based on multilevel meta-regression models to assess whether the measured anatomical region, mean patient age, and male/female ratio in each study were associated with IENFD. We applied this approach rather than the usual meta-regression method to consider the potential correlation between multiple outcomes from the same survey. The Stata Multilevel Mixed-Effects Reference Manual, Release 13, describes the corresponding methods. A *p*-value of <0.05 was considered to indicate statistical significance. Statistical analyses were performed using the statistical package STATA version 13.1 (STATA Corp., College Station, TX, USA).

### 2.4. Study Identification

**Figure 1:**
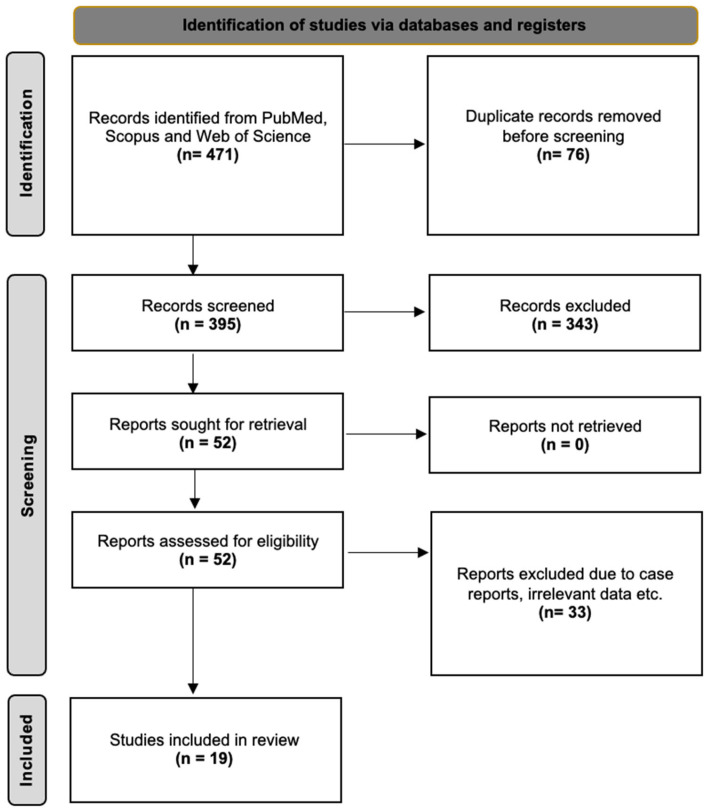
The flow chart of the search analysis.

A total of 471 papers, including case reports, clinical studies, reviews, letters to the editor, and conference abstracts, were identified through database searching and additional review of the reference lists. We excluded 452 articles due to irrelevance, incomplete data, lack of data after qualitative analysis, methodological differences (i.e., the immunofluorescence process), and insufficient numeric data regarding IENFD in limbs. We limited articles to those written in English and published during the last 25 years (1997–2022) because earlier clinical studies were based on qualitative criteria. The final sample for this meta-analysis included 19 studies (Figure 1).

## 3. Results

### 3.1. Study Population

The current meta-analysis included 863 healthy (control group) and 304 neuropathic adult subjects. The neuropathic backgrounds included diabetes mellitus, Friedreich ataxia, vasculitis, idiopathic neuropathy, sensory ganglionopathy, symmetrical sensory polyneuropathy, sensory neuropathy in systemic lupus erythematosus, neuropathy associated with chronic inflammatory demyelination, neuropathy in sarcoidosis, axonal swelling, HIV neuropathy (symptomatic and asymptomatic), and Charcot-Marie-Tooth disease type 1A (Table 1).

Table 2 summarizes the mean IENFD values and the corresponding SD and CV% values in healthy and neuropathic subjects. The majority of investigators conducted measurements on the distal leg (21 studies in healthy subjects and 14 in neuropathic subjects) and thigh (nine studies in healthy subjects and eight in neuropathic subjects). Thirty-six (36) studies focused on healthy populations, compared to 26 studies addressing neuropathic populations. Higher IENFD values were detected in the thigh, followed by the forearm, fingers, and toes. Compared to neuropathic subjects, healthy individuals had slightly higher SD values (median 4.7 vs. 3.5) and markedly higher mean IENFD (median 14.4 vs. 6.5). Thus, CV values (i.e., the relative dispersion, expressed as the quotient SD/mean) were higher in neuropathic patients (median 54.5 vs. 31.5), indicating that IENFD values in the neuropathic population were less consistent.

The thigh was preferred as the reference region because it had a lower CV (23.8%) in healthy subjects compared to the other tested anatomical areas, except for the toes, due to the particularly small sample (*n* = 2). The thigh was also used as the reference anatomical region in neuropathic patients, with a CV of 40.8%.

In summary, Table 2 highlights that the thigh IENFD is relatively preserved in early/SFN, making it suitable for detecting subclinical changes. The forearm and distal leg exhibit the most considerable absolute and relative differences, rendering them ideal for diagnostic use. Toes demonstrate the highest coefficient of variation in neuropathic patients (CV ~82%), indicating inconsistent fiber loss and limited utility without strict standardization. Overall, neuropathic groups display both lower median IENFD and higher variability, consistent with disease progression and diverse etiologies.

**Figure 2:**
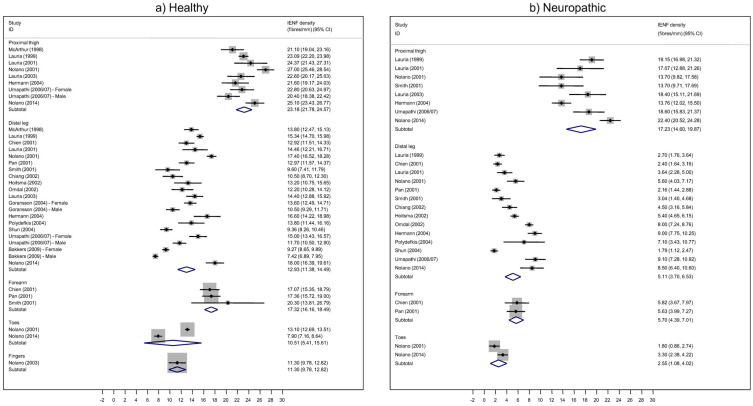
Forest plots of intraepidermal nerve fiber density (IENFD) by anatomical location. (**a**) Healthy subjects (control group) [3,15,18,19,20,21,22,23,25,27,28,29,30,31,32,33,35,36,37,38]. (**b**) Patients with neuropathy. Neuropathic patients consistently show a 50–80% reduction in IENFD across all biopsy sites [3,15,18,19,20,21,22,23,25,27,28,29,30,31,32,33,35,36,37,38]. Distal regions (toes and distal leg) exhibit the most pronounced nerve fiber loss. Forest plots reveal tight confidence intervals in healthy subjects and more heterogeneity in neuropathic groups, reflecting disease variability. These values align well with clinical diagnostics for small fiber neuropathy, especially in skin biopsy interpretation.

Using standard meta-analysis techniques, we determined the pooled estimates of mean IENFD and the corresponding confidence intervals (CI) per anatomical region and overall in healthy (Figure 2a) and neuropathic (Figure 2b) individuals. Among healthy subjects, the pooled estimate of mean IENFD in the forearm was 17.3 fibers/mm (95% CI: 16.2, 18.5), based on three studies (a marginally acceptable number of studies from which to extract an ES). These studies did not exhibit significant heterogeneity (*p* = 0.639; I^2^ = 0%). Unique research showed an IENFD in fingers of 11.3 fibers/mm (95% CI: 9.8, 12.8). The pooled estimate of mean IENFD in the thigh was 23.2 fibers/mm (95% CI: 21.8, 24.6), with statistically significant heterogeneity (*p* < 0.001; I^2^ = 81%). The corresponding estimates in the distal leg were 12.9 fibers/mm (95% CI: 11.4, 14.6) with very high heterogeneity (*p* < 0.001; I^2^ = 97.4%). In three studies that provided distinct data from males and females, the IENFD values for the distal leg were higher in females than in males. From only two studies, the pooled mean IENFD in toes was 14.5 fibers/mm (95% CI: 10.5, 15.6). Among neuropathic patients, the pooled estimated mean IENFD in the forearm was 5.7 fibers/mm (95% CI: 4.4, 7.0). The corresponding estimate in the thigh was 17.2 fibers/mm (95% CI: 14.6, 19.9) with high heterogeneity (*p* < 0.001; I^2^ = 87%). The pooled mean IENFD in the distal leg was 5.1 fibers/mm (95% CI: 3.7, 6.5), and in toes was 2.5 (95% CI: 1.1, 4.0), based on only two studies.

### 3.2. Multilevel Meta-Regression Results: Anatomical Region

We employed a multilevel meta-regression model to examine IENFD differences across anatomical regions in healthy subjects and neuropathic patients. The distal leg was used as the reference point since most measurements among the studies were taken from this location (Table 3).

For the healthy population, the estimated IENFD values were 17.7 fibers/mm in the forearm, 11.3 fibers/mm in the fingers, 21.4 fibers/mm in the thigh, 12.9 fibers/mm in the distal leg, and 6.50 fibers/mm in the toes (*p* < 0.001).

For the neuropathic population, the estimated IENFD values were 6.3 fibers/mm in the forearm, 17.2 fibers/mm in the thigh, 5.1 fibers/mm in the distal leg, and 2.0 fibers/mm in the toes.

**Figure 3:**
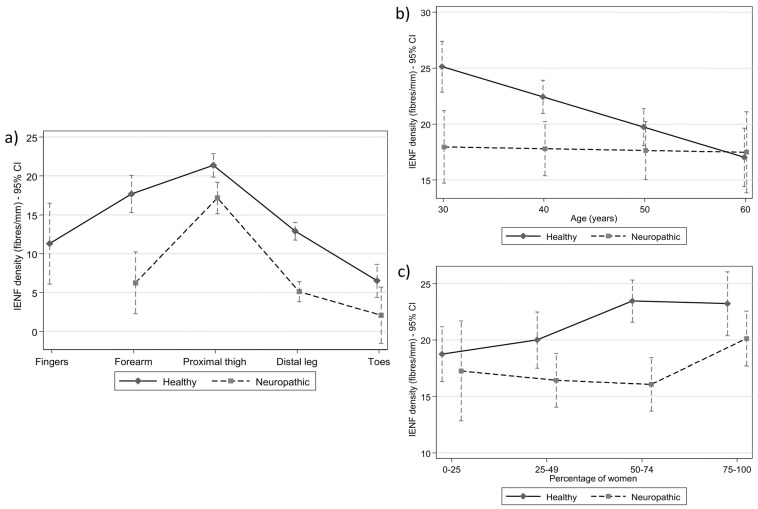
Estimated mean intraepidermal nerve fiber density (IENFD) by (**a**) anatomical location, (**b**) mean age, and (**c**) proportion of women in healthy subjects and neuropathic patients. Bars indicate 95% confidence intervals (CIs).

A control was performed to account for any possible interaction between the measurement and reference point for coefficient determination (distal leg) in both healthy subjects and neuropathic patients. The IENFD values were higher proximally and lower distally (Figure 3a). Neuropathic patients had lower mean IENFD values than healthy subjects in the same anatomical regions. The estimated difference in IENFD between the neuropathic and healthy populations was −7.95 (95% CI: −9.16, 6.75) (*p* < 0.001). Among healthy subjects, the IENFD of the reference point (distal leg) significantly differed from the IENFD of the forearm (coefficient, 4.8; *p* < 0.001), the thigh (8.5; *p* < 0.001), and the fingers (−1.6; *p* < 0.001) (Table 3). Among neuropathic patients, the IENFD of the distal leg significantly differed from the IENFD of the thigh (coefficient, 12.1; *p* < 0.001), but it did not substantially differ from the forearm (+1.1; *p* = 0.582) or the toes (−3.0; *p* = 0.117). The estimations for the fingers and forearm were inconsistent, with a large CI due to the limited data for these anatomical regions.

All estimates and their 95% CIs are shown in Figure 3a. Differences across anatomical regions were similar in both groups (interaction *p*-value = 0.084), with the overall difference between neuropathic patients and healthy individuals being −8.0 (95% CI: −9.2, −6.8; *p* < 0.001) fibers/mm.

### 3.3. Multilevel Meta-Regression Results: Age

Multilevel meta-regression analysis was also used to analyze the differences in IENFD according to age among healthy and neuropathic individuals, adjusting for differences across anatomical regions. Among healthy subjects, the mean IENFD was estimated to decrease by 1.35 fibers/mm (95% CI: −2.01, −0.69) every 5 years (*p* < 0.001). The corresponding estimate in the neuropathic population was a much slower decrease of 0.1 fibers/mm (95% CI: −0.8, 0.9) per 5 years, which, in contrast with healthy subjects, was not significant (*p* = 0.851). The difference between the two decreased rates was statistically significant (interaction *p*-value = 0.084). The estimated leg IENFD mean values and their 95% CIs at different ages in healthy individuals and neuropathic patients are shown in Figure 3b.

### 3.4. Multilevel Meta-Regression Results: Gender

Finally, we applied multilevel meta-regression analysis to examine IENFD differences according to the percentage of women in each study in healthy and neuropathic populations, adjusting for differences across anatomical regions. The percentage of women was entered as a categorical variable with four levels: group 1, 0–25% females; group 2, 25–49% females; group 3, 50–74% females; and group 4, 75–100% females.

Within the healthy population, the estimated leg IENFD value was 9.9 (95% CI: 7.8, 11.9) for group 1 and higher by 1.26 (95% CI: −1.7, 4.2; *p* = 0.399), 4.71 (95% CI: 2.3, 7.2; *p* < 0.001) and 4.5 (95% CI: 1.2, 7.7; *p* = 0.007) units in groups 2, 3 and 4, respectively. In the neuropathic population, the estimated leg IENFD value was 5.4 (95% CI: 1.5, 9.3) for group 1. Differences of the remaining groups, relative to group 1, were not significant: −0.8 (95% CI: −5.2, 3.6; *p* = 0.711), −1.2 (95% CI: −5.4, 3.0; *p* = 0.580) and 2.9 (95% CI: −1.5, 7.3; *p* = 0.202) for groups 2, 3 and 4, respectively. The estimated leg IENFD mean values and their 95% CIs for the four groups in healthy individuals and neuropathic patients are shown in Figure 3c. In summary, Figure 3 indicates that neuropathy flattens the normal IENFD distribution across different sites and ages. IENFD loss is most pronounced in the distal extremities. Aging and sex significantly influence IENFD in healthy individuals, but not in those with neuropathy, highlighting the dominant impact of disease-related factors over physiological ones.

## 4. Discussion

Skin spot biopsy is the gold standard for diagnosing small fiber neuropathy, and IENFD decrease is correlated with neuropathy [1].

### 4.1. IENFD Distribution Among Studies and Anatomical Regions from Proximal to Distal

The current meta-analysis aimed to determine estimated IENFD values in healthy subjects, creating a guidance model for distinguishing normal from pathological values. Among the reviewed studies, the majority of measurements were taken from the distal leg [11,39,40,41], and most studies show a proximal-to-distal gradient of IENFD values in normal subjects, with IENFD at the distal thigh being about 60% higher than at the distal leg [36]. The most consistent measurement point was the thigh, which showed the lowest CI (23.8%). However, the lack of estimated IENFD values in healthy populations impedes direct comparisons, and the impacts of age and gender on IENFD in healthy individuals remain controversial [39].

IENFD decreased from proximally to distally across anatomical measurement regions and differed between the upper and lower limbs. In the healthy population, the estimated IENFD values in order of decreasing frequency were 21.4 fibers/mm for the thigh, 17.79 fibers/mm for the forearm, 12.9 fibers/mm for the distal leg, 11.3 fibers/mm for the fingers, and 6.5 fibers/mm for the toes. We observed broad heterogeneity between measurements at the same anatomical region within the same study. This phenomenon may be explained by the lack of homogeneity between skin points—for example, in the case of pocket follicles and sweat glands, which show variable densities.

### 4.2. IENFD Values and Gender Impact

We observed gender dimorphism in IENFD, with females exhibiting higher values in the distal leg (13.6–10.5 fibers/mm) compared to males (9.3–7.2 fibers/mm). Females also seemed somewhat resistant to the IENFD reduction due to neuropathy, but this phenomenon remains unclear. Goransson et al. [38] found higher IENFD values in females than in males.

### 4.3. IENFD Values and Age Impact

In a healthy population, IENFD decreased with aging, with a 1.35 fiber/mm reduction every five years. Several authors have reported higher IENFD values in younger individuals [36], while others describe IENFD reduction with aging [16,24,38].

### 4.4. IENFD Values and Neuropathy Impact

IENFD rapidly decreased in patients with neuropathic disease, exhibiting a consistent alteration over the transition from a healthy status to illness. We found that a person with neuropathy (independently of age) has the same IENFD as a healthy elder. This finding supports the notion that the substantial decrease in SNFs observed in the neuropathic population may be due to the transition from a healthy status to pathology. This effect strongly suggests that the IENFD alteration may be a helpful index with high predictive value.

Our meta-regression analysis showed that the mean IENFD differed based on the proportion of females in the healthy population. Among the healthy participants, an increased percentage of women was associated with a higher IENFD (systematic trend). These differences were statistically significant, and the direction aligned with the hypothesis that women have higher IENFD values (Figure 3c). In contrast, within the population of neuropathic patients, the meta-regression analysis did not reveal a clear trend of IENFD differences according to the proportion of neuropathic women. Instead, this analysis indicated significant differences in only the last two categories (50–74% females and over 75% females). These findings may suggest that women suffering from neuropathy have greater resistance to the loss of SNFs; however, no systematic trend was detected.

### 4.5. IENFD Values Variability and Heterogeneity—Contributing Factors and Limitations

**Figure 4:**
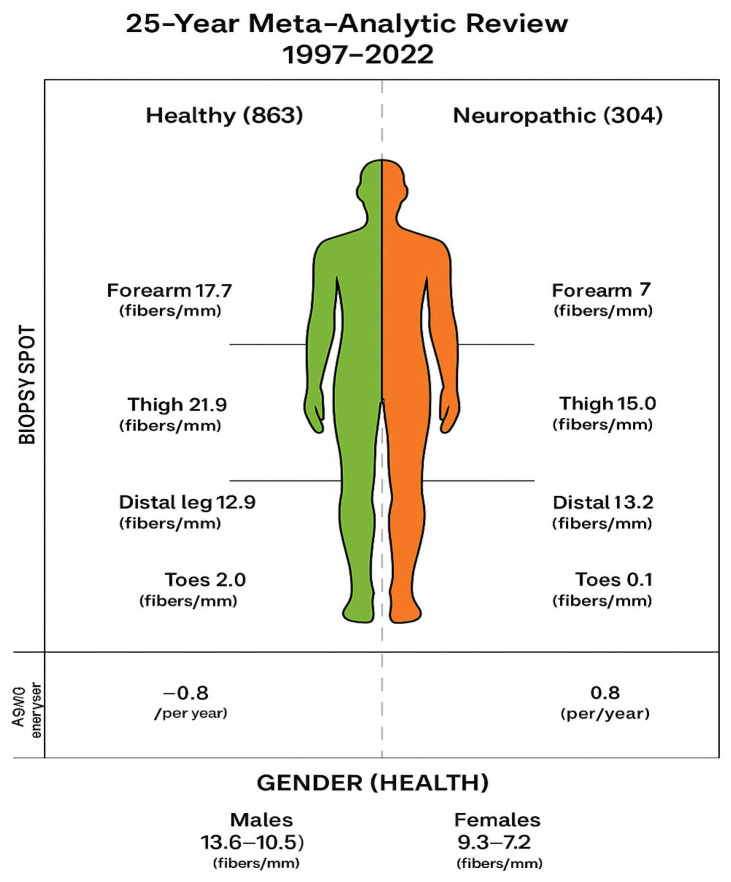
A schematic representation of the current meta-analysis results, emphasizing the gender impact of intraepidermal nerve fiber density (IENFD) across various anatomical regions in both healthy and neuropathic subjects.

This analysis employs meta-regression techniques to address inter-study variability attributed to factors such as biopsy site, fixation protocols, immunohistochemical markers, and patient demographics (Figure 4). Additionally, there is considerable heterogeneity among the group of neuropathic patients, with a variety of diseases represented. We recognize this as a limitation of our study. However, because of the limited number of studies focusing on each of the neurodegenerative diseases included in our research, we decided to combine them to achieve reasonable precision and power in our analyses. Numerous technical, biological, and analytical factors may influence IENFD measurements, thereby introducing bias or variability in research and clinical diagnoses, such as SFN. Technical considerations, including biopsy site, depth, fixation, tissue handling, section thickness, staining protocol, antibody quality, and microscope calibration, can all affect procedural bias. Furthermore, patient-specific variables, including age, sex, ethnicity, skin pigmentation, and disease states, must also be taken into account. Additionally, counting bias and interpretation errors, such as subjective variability in manual counting, definitions of “fiber crossing the dermal-epidermal junction”, edge effects, and sampling bias, which restricts counting to the central region of a section to mitigate edge effects, can significantly impact measurements.

### 4.6. IENFD Decrease Values and the Relationship Among Healthy Status and Neuropathy Disease

Reduced IENFD is a hallmark of SFN, reflecting shared mechanisms across neurodegenerative and systemic diseases. Central to this is the degeneration of unmyelinated (C-fibers) and thinly myelinated (Aδ-fibers) axons due to direct neuronal injury, mitochondrial dysfunction, and inflammatory damage [42]. Conditions such as diabetes mellitus, human immunodeficiency virus (HIV), systemic lupus erythematosus, and vasculitis impair axonal transport, resulting in distal axonopathy and a loss of epidermal innervation [43]. Mitochondrial dysfunction in Friedreich ataxia compromises energy metabolism, making small fibers susceptible to oxidative stress [44]. Chronic infections like HIV worsen this condition due to ongoing immune activation and neurotoxic cytokines such as TNF-α and IL-6 [12]. In inherited neuropathies like Charcot-Marie-Tooth disease type 1A (CMT1A), duplication of the PMP22 gene results in Schwann cell dysfunction and impaired axonal support [44]. Despite varying causes, these diseases result in selective SFNs damage, which can be detected through skin biopsy and IENFD quantification, even in the absence of large-fiber involvement as seen in nerve conduction studies [12].

## 5. Conclusions

The current systematic study, accompanied by a meta-analysis, thoroughly integrates evidence of IENFD in human skin biopsies. Over the course of two decades, this meta-analysis aims to reconcile findings derived from diverse methodologies and populations. By employing meta-regression techniques, this analysis addresses inter-study variability attributed to factors such as biopsy site, fixation protocols, immunohistochemical markers, and patient demographics. To reduce heterogeneity in future studies, it is advisable to use the thigh as the anatomical region, as it appears to exhibit the least heterogeneity among the studies. Furthermore, the precise biopsy site should be identified internationally to ensure comparability of all results. The findings necessitate additional investigations into the alterations of IENFD (either increases or decreases) in neurodegenerative diseases. Examining whether the IENFD value may serve as a reliable prognostic marker for the prompt diagnosis of neuropathy is essential.

## Figures and Tables

**Table 1:** The studies included in the meta-analysis. All figures refer to Healthy (H) and Neuropathic (N), respectively. M = males and F = females.

Studies by Authors [Citation]	Year	Status	Sample	Type of Neuropathy	Mean Age of H/N	H/N by Gender	
		H/N	*N*		Total	Females (%)	Males (%)
McArthur et al. [35]	1998	98/0	98	-	47.5/-	32.7/-	67.3/-
Lauria et al. [15]	1999	10/6	16	Sensory Fiber Neuropathy	49.0/56.5	50.0/66.7	50.0/33.3
Chien et al. [22]	2001	55/35	90	Cutaneous Nerve Terminal Degeneration	45.9/47.3	65.5/51.4	34.5/48.6
Lauria et al. [21]	2001	15/16	31	Sensory Ganglionopathies	NA/NA	NA/NA	NA/NA
Nolano et al. [18]	2001	51/14	65	Friedreich’s Ataxia	31.7/29.4	52.9/71.4	47.1/28.6
Pan et al. [19]	2001	55/35	90	Peripheral Neuropathy	45.9/47.3	65.5/51.4	34.5/48.6
Smith et al. [20]	2001	5/8	13	Impaired Glucose Tolerance, Diabetes Mellitus	NA/NA	NA/NA	NA/NA
Chiang et al. [36]	2002	18/18	36	Chronic Inflammatory Demyelinating Polyneuropathy	45.1/45.1	33.3/33.3	66.7/66.7
Hoitsma et al. [25]	2002	6/7	13	Sarcoidosis	35.0/36.0	50.0/14.3	50.0/85.7
Omdal et al. [23]	2002	15/15	30	Systemic Lupus Erythematosus	47.3/47.3	93.3/93.3	6.7/6.7
Lauria et al. [3]	2003	15/15	30	Axonal Swellings in Painful Neuropathies	NA/49.5	60.0/46.7	40.0/53.3
Nolano et al. [37]	2003	14/0	14	-	33.7/-	57.1/-	42.9/-
Goransson et al. * [38]	2004	40/0 M	106	-	57.5/-	0.0/-	100.0/-
Goransson et al. * [38]	2004	66/0 F		-	43.8/-	100.0/-	0/-
Hermann et al. [27]	2004	19/40	59	Axonal Swellings and QST in HIV Distal Sensory Neuropathy	34.4/42.0	NA/25.0	NA/75.0
Polydefkis et al. [29]	2004	31/8	39	Diabetes Mellitus	37.5/54.4	58.1/50.0	41.9/50.0
Shun et al. [28]	2004	38/38	76	Diabetes Mellitus	55.5/56.2	34.2/34.2	65.8/65.8
Umapathi et al. [30]	2007	0/29	29	Early Diabetic Neuropathy	-/48.4	-/24.1	-/75.9
Umapathi * [30]	2006	45/0 M	84	-	43.2/-	0.0/-	100.0/-
Umapathi * [30]	2006	39/0 F		-	35.1/-	100.0/-	0/-
Bakkers * [33]	2009	91/0 M	188	-	48.4/-	0.0/-	100.0/-
Bakkers * [33]	2009	97/0 F		-	51.6/-	100.0/-	0/-
Nolano et al. [31]	2014	40/20	60	Charcot-Marie-Tooth type 1A	44.2/42.9	75.0/75.0	25.0/25.0
All studies		863/304	1167				

* Data refers to male (Μ) and female (F) subjects rather than the total sample. *N*: number of healthy and neuropathic subjects; -: not applicable; NA: data not available.

**Table 2:** Distribution of mean intraepidermal nerve fiber density (IENFD in fibers/mm), standard deviation (SD), and coefficient of variation (CV) in healthy and neuropathic subjects across studies. All figures represent the median and interquartile range (IQR).

IENFD (Fibers/mm) in Different Areas Among Studies	Population			Observations by Anatomical Area
	Healthy (*n* = 36)	Neuropathic (*n* = 26)	Total (*n* = 62)	
	Median (IQR)	Median (IQR)	Median (IQR)	
Mean n (Healthy/Neuropathic among studies)				
Forearm (3/2)	17.4 (17.1, 20.3)	5.7 (5.6, 5.8)		Significant loss: stable median and SD suggest diagnostic clarity
Fingers (1/0)	11.3 (11.3, 11.3)	-		Limited data. Used occasionally
Thigh (9/8)	22.8 (21.6, 24.4)	17.7 (13.7, 18.9)		The least affected site in neuropathy
Distal leg (21/14)	13.2 (10.5, 14.5)	5.0 (2.7, 8.0)		Common biopsy site; large diagnostic gap
Toes (2/2)	10.5 (7.9, 13.1)	2.5 (1.8, 3.3)		Distal-most loss; very high variation
**Overall N (36/26)**	**14.4 (11.9, 20.4)**	**6.5 (3.3, 13.7)**	**13.1 (7.9, 17.4)**	
Standard Deviation (SD)				
Forearm	6.5 (6.2, 7.4)	5.7 (5.0, 6.5)		Significant loss: stable median and SD suggest diagnostic clarity
Fingers	2.9 (2.9, 2.9)	-		Limited data. Used occasionally
Thigh	5.6 (5.4, 6.9)	6.1 (5.0, 7.5)		The least affected site in neuropathy
Distal leg	3.9 (3.1, 5.2)	2.6 (2.1, 4.0)		Common biopsy site; large diagnostic gap
Toes	2.0 (1.5, 2.4)	2.0 (1.8, 2.1)		Distal-most loss; very high variation
**Overall**	**4.7 (3.1, 5.7)**	**3.5 (2.2, 5.6)**	**4.4 (2.7, 5.6)**	
Coefficient of variation (CV) (%)				
Forearm	36.5 (35.7, 38.1)	99.8 (88.0, 111.7)		Significant loss: stable median and SD suggest diagnostic clarity
Fingers	25.7 (25.7, 25.7)	-		Limited data. Used occasionally
Thigh	23.8 (21.2, 30.3)	40.8 (27.3, 46.1)		The least affected site in neuropathy
Distal leg	33.8 (28.9, 37.1)	60.5 (44.8, 78.0)		Common biopsy site; large diagnostic gap
Toes	20.9 (11.5, 30.4)	81.8 (63.6, 100.0)		Distal-most loss; very high variation
**Overall**	**31.5 (23.5, 36.7)**	**54.5 (40.9, 78.0)**	**36.1 (25.7, 50.1)**	

**Table 3:** Estimated IENFD (fibers/mm) differences relative to the reference point (distal leg) and values in healthy and neuropathic subjects. Results from multilevel meta-regression.

Healthy	Differences			Values	
Measurement Point	Estimate	95% CI	*p* Value	Estimate	95% CI
Distal leg				12.89	(11.75, 14.03)
Forearm vs. Distal leg	4.80	(2.57, 7.03)	<0.001	17.69	(15.30, 20.09)
Fingers vs. Distal leg	−1.59	(−6.93, 3.74)	0.558	11.30	(6.09, 16.51)
Thigh vs. Distal leg	8.49	(7.25, 9.73)	<0.001	21.38	(19.87, 22.90)
Toes vs. Distal leg	−6.39	(−8.34, −4.45)	<0.001	6.50	(4.37, 8.64)
**Neuropathic**	**Differences**			**Values**	
**Measurement Point**	**Estimate**	**95% CI**	***p*** **Value**	**Estimate**	**95% CI**
Distal leg				5.11	(3.81, 6.41)
Forearm vs. Distal leg	1.14	(−2.93, 5.22)	0.582	6.26	(2.28, 10.23)
Thigh vs. Distal leg	12.06	(9.75, 14.36)	<0.001	17.17	(15.15, 19.18)
Toes vs. Distal leg	−3.04	(−6.84, 0.76)	0.117	2.07	(0.00, 5.69)

## Data Availability

All the data are available to the corresponding author upon reasonable request.
